# Ancient DNA reveals monozygotic newborn twins from the Upper Palaeolithic

**DOI:** 10.1038/s42003-020-01372-8

**Published:** 2020-11-06

**Authors:** Maria Teschler-Nicola, Daniel Fernandes, Marc Händel, Thomas Einwögerer, Ulrich Simon, Christine Neugebauer-Maresch, Stefan Tangl, Patrick Heimel, Toni Dobsak, Anika Retzmann, Thomas Prohaska, Johanna Irrgeher, Douglas J. Kennett, Iñigo Olalde, David Reich, Ron Pinhasi

**Affiliations:** 1 Department of Anthropology, Natural History Museum Vienna, A-1010 Vienna, Austria; 2 Department of Evolutionary Anthropology, University of Vienna, A-1090 Vienna, Austria; 3 CIAS, Department of Life Sciences, University of Coimbra, 3000-456 Coimbra, Portugal; 4 Institute for Oriental and European Archaeology, Austrian Academy of Sciences, A-1020 Vienna, Austria; 5 Karl Donath Laboratory for Hard Tissue and Biomaterial Research, University Clinic of Dentistry Vienna, Medical University of Vienna, A-1090 Vienna, Austria; 6 Austrian Cluster for Tissue Regeneration, A-1200 Vienna, Austria; 7 Ludwig Boltzmann Institute for Experimental and Clinical Traumatology, AUVA Research Center, A-1200 Vienna, Austria; 8 Chair of General and Analytical Chemistry, Montanuniversität Leoben, A-8700 Leoben, Austria; 9 Department of Anthropology, University of California, Santa Barbara, CA 93106 USA; 10 Department of Genetics, Harvard Medical School, Boston, MA 02115 USA; 11 Broad Institute of Harvard and MIT, Cambridge, MA 02142 USA; 12 Howard Hughes Medical Institute, Harvard Medical School, Boston, MA 02115 USA; 13 Department of Human Evolutionary Biology, Harvard University, Cambridge, MA 02138 USA

**Keywords:** Genomics, Anthropology

## Abstract

The Upper Palaeolithic double burial of newborns and the single burial of a ca. 3-month-old infant uncovered at the Gravettian site of Krems-Wachtberg, Austria, are of paramount importance given the rarity of immature human remains from this time. Genome-wide ancient DNA shows that the male infants of the double grave are the earliest reported case of monozygotic twins, while the single grave´s individual was their 3rd-degree male relative. We assessed the individuals´ age at death by applying histological and µCT inspection of the maxillary second incisors (i2) in conjunction with C- and N-isotope ratios and Barium (Ba) intake as biomarker for breastfeeding. The results show that the twins were full-term newborns, and that while individual 2 died at birth, individual 1 survived for about 50 days. The findings show that Gravettian mortuary behaviour also included re-opening of a grave and manipulation of its layout and content.

## Abstract (web summary)

Maria Teschler-Nicola et al. use ancient DNA sequencing to report the earliest known case of human monozygotic twins found in a previously discovered Upper Palaeolithic burial site. Using bioanthropological and archaeological techniques, they also find that the twins were full-term newborns and that ancient mortuary behavior included re-opening of grave sites to bury related individuals together.

## Introduction

**Fig. 1:**
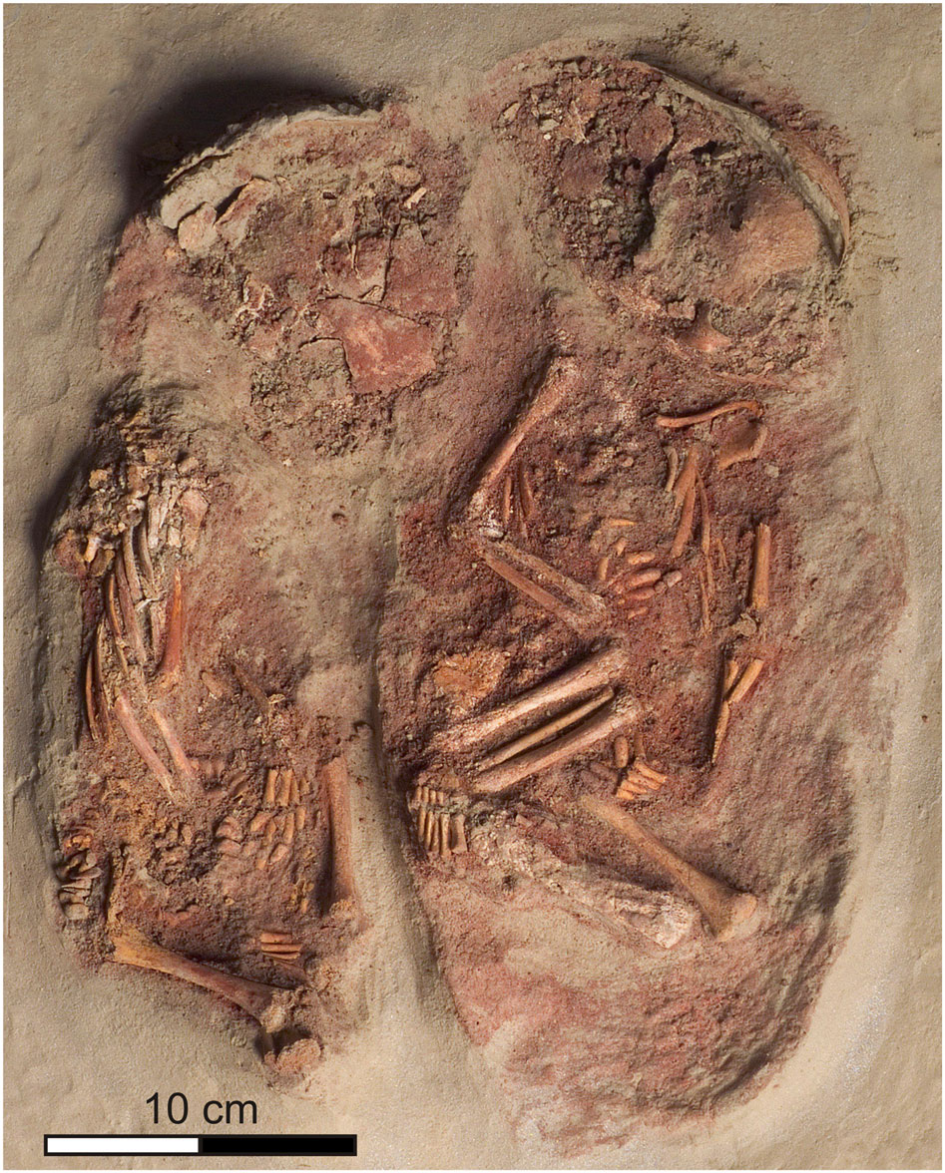
Krems-Wachtberg (Austria). Burial 1 with the skeletal remains of two infants recovered as block in 2005 (ind1 on the left, ind2 on the right). Photograph: Natural History Museum Vienna; modified.

**Fig. 2:**
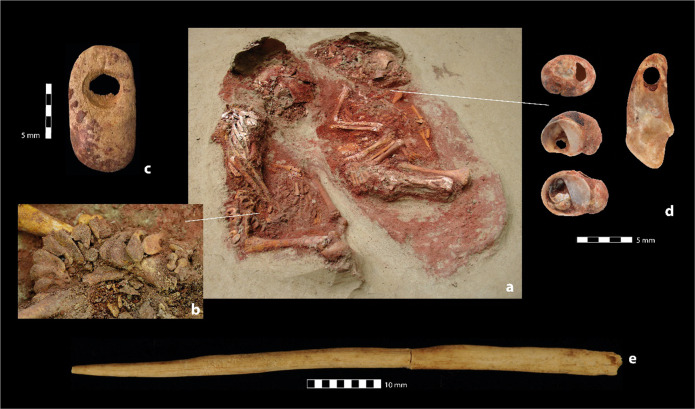
Burial context and findings. **a** The twin’s bodies (individual 1 and 2) in the grave pit of Burial 1. **b**, **c** Mammoth ivory beads and their arrangement on individual 1’s pelvis. **d** Adornment of Individual 2 consisting of a perforated fox incisor (*Vulpes* sp.) and three perforated molluscs (*Theodoxus* sp.). **e** Ivory pin from Burial 2 (individual 3) (find numbers: **c** Ivory bead WA-18158; **d** molluscs (from top to bottom) WA-151565, WA-151561, WA-151564, fox incisor WA-151558; **e** ivory pin WA-37552). Photographs: OREA, Austrian Academy of Sciences. Graph: R. Thomas.

Between ~40,000 and 30,000 years ago, mobile Upper Palaeolithic hunter–gatherer groups repeatedly occupied the promontory on the Danube’s left bank above what is today the town centre of Krems, Lower Austria^1,2^. The extensive archaeological remains of these occupations, which are embedded in thick loess sediment sequences, play a significant role in understanding settlement patterns, culture, and economy of early anatomically modern humans in Central Europe. Among these Upper Palaeolithic find spots, Krems-Wachtberg stands out due to exceptional preservation of organic materials and occupation structures^3^. Here, buried beneath more than 5 m of loess sediment, archaeological excavations revealed an occupation floor extending over ca. 45 m^2^ displaying an ensemble of well-preserved interrelated settlement structures that allow for detailed insights into Upper Palaeolithic hunter–gatherer behaviour, including a large multi-functional hearth with connected pits, and two infant burials^4,5^. Typological and technological characteristics of the artefacts, which include art objects and personal adornments, together with economic and socio-cultural criteria attribute this occupation to the Pavlovian, a regional expression of the earlier Gravettian^6–8^. Radiocarbon determinations average at 31,000 cal bp (IntCal13) resp. 31,700 cal bp (CalPal 2007 HULU)^9,10^. This agrees with the chronostratigraphic placement of the occupation into a phase of climatic cooling and onset of growing ice sheets during the approach of the Last Glacial Maximum^11,12^. Seasonality data provided by the faunal remains of hunted prey indicate that the camp was used during winter or early spring^13^. A Gravettian/Pavlovian attribution of the occupation is also attested for the infant burials which are of deep interest for ontogenetic studies of early anatomically modern humans given the rarity of immature human skeletal remains of the Upper Palaeolithic^14–17^. They also substantially enrich the debate about ritualistic and mortuary practices among Gravettian hunter–gatherer societies. The oval-shaped grave pit of the double burial (Burial 1), measuring ca. 0.36 × 0.28 × 0.2 m (length × width × depth) contained the skeletal remains of two infants (individuals ind1 and ind2, Fig. 1). Each of the bodies was embedded in red ochre and they were placed next to each other in flexed positions facing east and with their skulls pointing north^18^. The bodies, however, did not occupy the same amount of space in the grave pit: while ind2 was placed more centrally, ind1 was deposited against the grave pit’s southwest edge. A total of 53 beads made of mammoth ivory were set on ind1’s pelvis and their arrangement clearly indicate they had been threaded on a string (Fig. 2b). All 53 beads are remarkably similar in size and shape and the perforations show no sign of use-wear indicating a production for the sole purpose of serving as grave-good (Fig. 2c). Personalised attribution is emphasised by the position of the individual’s right hand which was placed atop the string. In contrast, ind2 was equipped with three perforated molluscs (*Theodoxus* sp.) and a perforated fox incisor (*Vulpes* sp.) that were recovered from beneath ind2’s mandible, suggesting they were pendants on a single necklace (Fig. 2d). After deposition of the corpses, the pit was not backfilled but instead sealed with a mammoth’s shoulder blade of which, to make it fit, the *spina scapulae* had been flaked off with a series of blows. One side of the scapula rested on the fragment of a mammoth’s tusk. As the grave pit had not been backfilled, an up to 7 cm deep space remained empty beneath the mammoth scapula, and only a fine layer (1–3 mm) of washed silt was deposited on the human remains and the red ochre. The longer and narrower grave pit of Burial 2, a single grave measuring ca. 0.45 × 0.22 × 0.15 m (length × width × depth), was found merely one and a half metres north of the double burial. It contained the poorly preserved skeleton of a further infant (ind3) which was interred in a flexed position and covered by a thick layer of red ochre. The child’s skeleton was also facing east, in this case, however, with its skull pointing south. In orientation of the body axis, an 8 cm long pin made of mammoth ivory (Fig. 2e) was located 2 cm above the skull. It may have been used as a cloakpin to fasten and/or decorate a garment, such as leather or a fur that had been wrapped around the body prior to burial. The use of a wrap is also suggested by the spindle-shaped boundary of the powdered red ochre. In contrast to Burial 1, this grave was not covered by protective elements, and the grave pit was backfilled. This most likely led to the poorer bone preservation of ind3 compared to ind1 and ind2 of Burial 1.

The infants of the double burial revealed a similar developmental stage (and assumingly perinatal death) which, together with the fact that they shared one grave, suggests that they were either twins or at least closely related individuals^4^. Although potential cases of twins have been documented in the archaeological record^19–21^, the arguments used to substantiate a twin status, have been consistently weak and were not able to provide conclusive support. In most of these cases, identification was based on physical similarity (e.g., tooth morphology) or features regarding the burial contexts. Only two cases are known from an archaeological context, where the exceptionally rare phenomenon of a twin status is unambiguously confirmed: one by dystocia^22^ and the second by discovery of foetal skeletal remains in utero^23^. All these studies failed in aDNA analysis due to insufficient preservation of the skeletal remains and/or aDNA.

The archaeological layout of the double burial suggests that the inhumation of the two individuals was not simultaneous (at the same time sensu stricto) but rather suggests that placement of the two bodies occurred in separate, consecutive steps. This would imply that the grave had been re-opened. Archaeological evidence for this possibility is provided by the dissimilar positions of the infants’ skeletons, the different dimensions of space occupied by the bodies (the grave seems to have been constructed for ind2, which occupies the main space, while ind1 appears huddled against the grave pit’s edge), as well as differential symbolic treatment displayed by the personalised adornments.

In this study we aim (i) to assess the genetic relation of the two infants of Burial 1 to establish whether kinship was the motivating factor behind their burial in a joint grave, (ii) to estimate the age at death for each individual to determine the time that could have elapsed between placing the infants’ corpses into the grave in order to provide an explanation for their divergent positions in the grave and their differential symbolic treatment, and (iii) to address ind3 (Burial 2) in the analyses for comparison and to complement our knowledge about mortuary und ritualistic practices of Gravettian hunter–gatherer communities.

## Results

### Anthropological assessment

#### Ancient DNA

To assess the sex and biological relationships of the three individuals we sampled their crania. We were able to obtain well-preserved endogenous DNA from a cranial vault fragment of ind3, the results of which have already been published^24^. In the case of ind1 and ind2, we sampled their petrous bones. After enriching for 1,240,000 single-nucleotide polymorphisms (SNPs), we obtained 722,470 SNPs on chromosomes 1–22 (1.772× average coverage) for ind1 by pooling data from two non-UDG-treated libraries (each SNP with a coverage of at least one sequence) (Table 1 and Supplementary Data 1). We recovered 264,795 SNPs (0.282× coverage) for ind2 by pooling data from four UDG-treated libraries (Table 1 and Supplementary Data 1). Both individuals in Burial 1 were consistent with being genetic males based on the ratio of sequenced reads aligning to the X and Y chromosomes (Table 1). Low contamination estimates (0–1.353%) and high deamination frequencies (ind1: 29.6%; ind2: 10.6%) support the authenticity of the recovered sequences (Table 1).

**Table 1:** Summary of sequencing data.

Archaeological ID	Lab ID	Average date range	Bone	Genetic sex	Average deamination frequency at 5′ end	Endogenous fraction	Mitochondrial Haplo-group	Y Haplo-group	1240K capture SNPs	1240K capture coverage	Mitochondrial contamination estimate	X chromosome contamination estimate [*Z*-score]
Krems1_1	I2483	30,950 cal bp (IntCal13) −31,750 cal bp (CalPal 2007 HULU)	Petrous	XY	0.296	0.030	U5	I	722,470	1.722	0.002	0.012 [2.282]
Krems1_2	I2484	30,950 cal bp (IntCal13) −31,750 cal bp (CalPal 2007 HULU)	Petrous	XY	0.106	0.011	U5	I	264,795	0.282	0.000	0.013 [0.740]

**Fig. 3:**
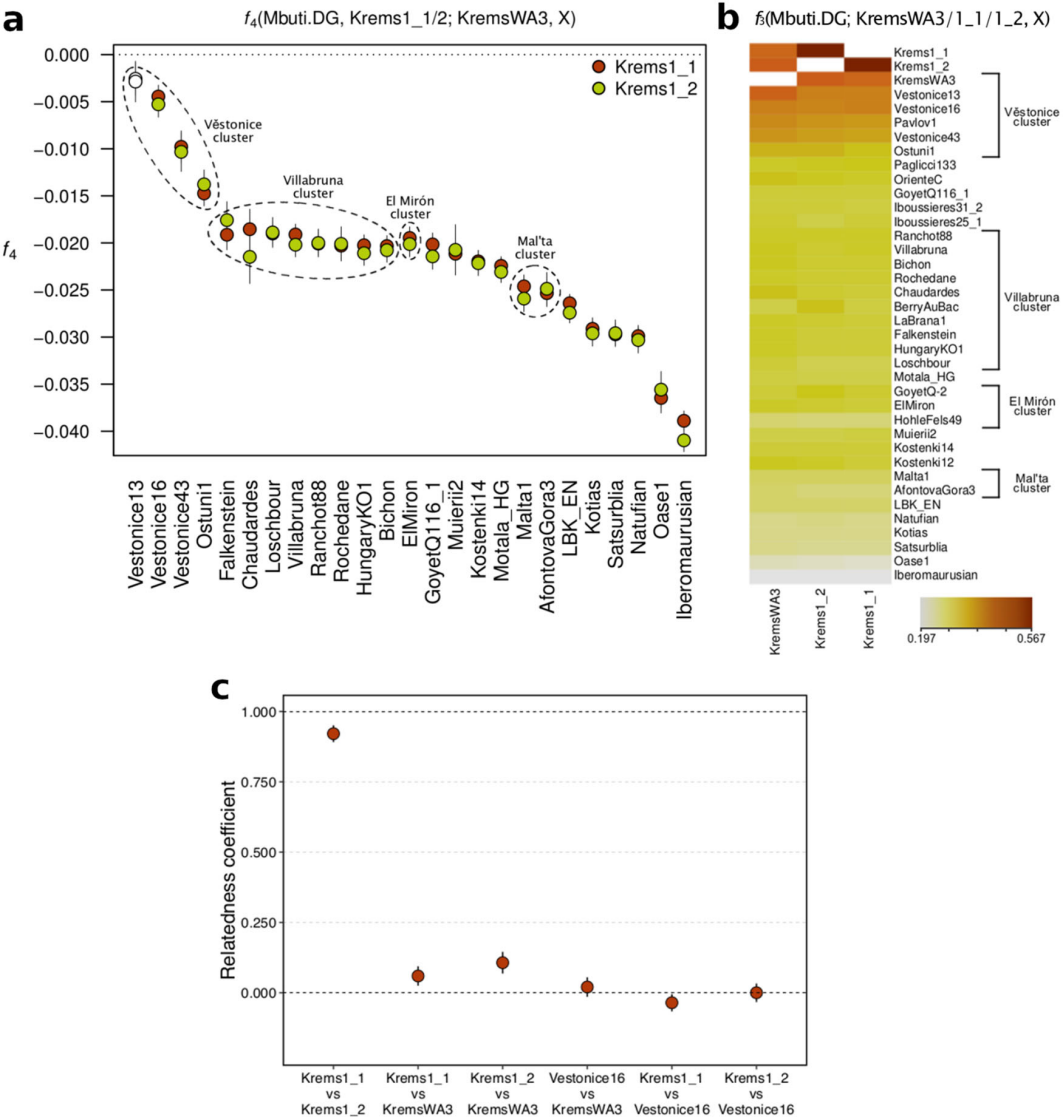
Population genetics relationship between Krems-Wachtberg and other Upper Palaeolithic and Mesolithic individuals and clusters, as defined by Fu et al.^24^. **a** Four-population symmetry tests showing that the individuals from Burial 1 (Krems1_1, Krems1_2) shared more alleles with ind3 (KremsWA3) from Burial 2 than to other tested individuals (white circles represent results that are not significant at a threshold of *Z* < |3|; the statistic for Věstonice13 falls below this threshold). **b** Outgroup-*f*_3_ results measuring pairwise shared genetic drift and showing the proximity of Krems-Wachtberg to the Věstonice cluster. **c** Kinship analysis results using Věstonice16 to calculate mismatch rates. The mismatch rate between KremsWA3 and the Krems twins is 1/8 to 1/16 of the way from the mismatch rates observed between Věstonice16 and the three Krems individuals, and zero mismatch, consistent with their being third- or fourth-degree relatives. Bars represent 95% confidence intervals.

To assess kinship and genetic affinities we carried out population genetic analyses of the three individuals together with previously published Eurasian Upper Palaeolithic and Mesolithic individuals. First, we analysed ind1 and ind2 using *f*_3_- and *f*_4_-statistics. The individuals have genetic affinities similar to those described by Fu et al. for ind3 (ref. ^24^). All three Krems-Wachtberg individuals share most alleles with each other, and then with individuals from the Gravettian population cluster named after the site of Dolní Věstonice (Czech Republic, ~100 km northeast)^24^ (Fig. 3a/b and Supplementary Data 2). The Burial 1 boys share significantly more alleles with ind3 than they do with any other Upper Palaeolithic/Mesolithic specimens analysed, except for Věstonice13 for whom the signal is non-significant (*Z* = −1.477, Fig. 3a and Supplementary Data 2), which points at close genetic ties between individuals from the two contemporaneous sites. Consequently, we used the Dolní Věstonice individuals to assess the Krems-Wachtberg infants’ kinship by calculating intra-population SNP mismatch rates for unrelated individuals following Olalde et al.^25^. Results show that the Burial 1 infants were identical twins sharing their entire genome, while ind3 was probably a third degree, or more distant, relative as the pairwise test between ind2 and ind3 resulted in a higher and non-overlapping relatedness coefficient than between any Krems-Wachtberg individual and the high-coverage individual Věstonice16 (Fig. 3c). The three Krems-Wachtberg individuals also shared the same major Y chromosome and mitochondrial haplogroups.

#### Morphometric assessment

The differential symbolic treatment and deposition of the twins suggested a possible non-synchronous interment related to different ages at death. Since stratigraphic observations were not able to unambiguously determine the chronological sequence of events, an estimation of their ages at death had to be considered the key indication. In most cases, the age at death of infants is determined based on metrical comparisons of long bone diaphyseal lengths and dental developmental stages. Although, on first view, the postcranial remains of the twins impressed by their apparently good state of preservation (Figs. 1 and 2), a closer inspection revealed substance losses and erosion considerably limiting the potential for age estimation. Moreover, a comparison of the individuals’ developmental degree is impeded by the fact that sufficiently preserved corresponding limb bones are often missing. The sole exception are the tibiae that were well enough preserved to measure the tibial lengths (ind1 = 62.5 mm, ind2 = 63.0 mm). Based on these dimensions, we estimate the age at death to be between the 9th and 10th month post-conception and a body size of ca. 50.1 cm^26,27^. We abstained from taking further measurements due to pathological alteration (periostitis) apparent on the tibial shafts of ind1. Measurements taken from other skeletal elements show intra-individual variation as well as minor discordances in size between the individuals. The latter is a quite common finding in twins^28^. As mentioned, we observed a well pronounced layer of connective tissue at the medial surfaces of the tibiae caused by a severe inflammation of the periosteum (periostitis). Generally, the periosteum is more loosely bound to the underlying cortical bone in infants than in adults, and thus prone to separation and haemorrhages. These changes can be caused by inadequate intake of vitamin C. As vitamin C deficiency would normally take several months after birth before manifesting itself in the form of subperiosteal new bone formation^29^, doubts remain regarding such a diagnosis in a perinate. Nevertheless, the layer clearly represents a pathological change that could impair skeletal development and dimensions.

**Fig. 4:**
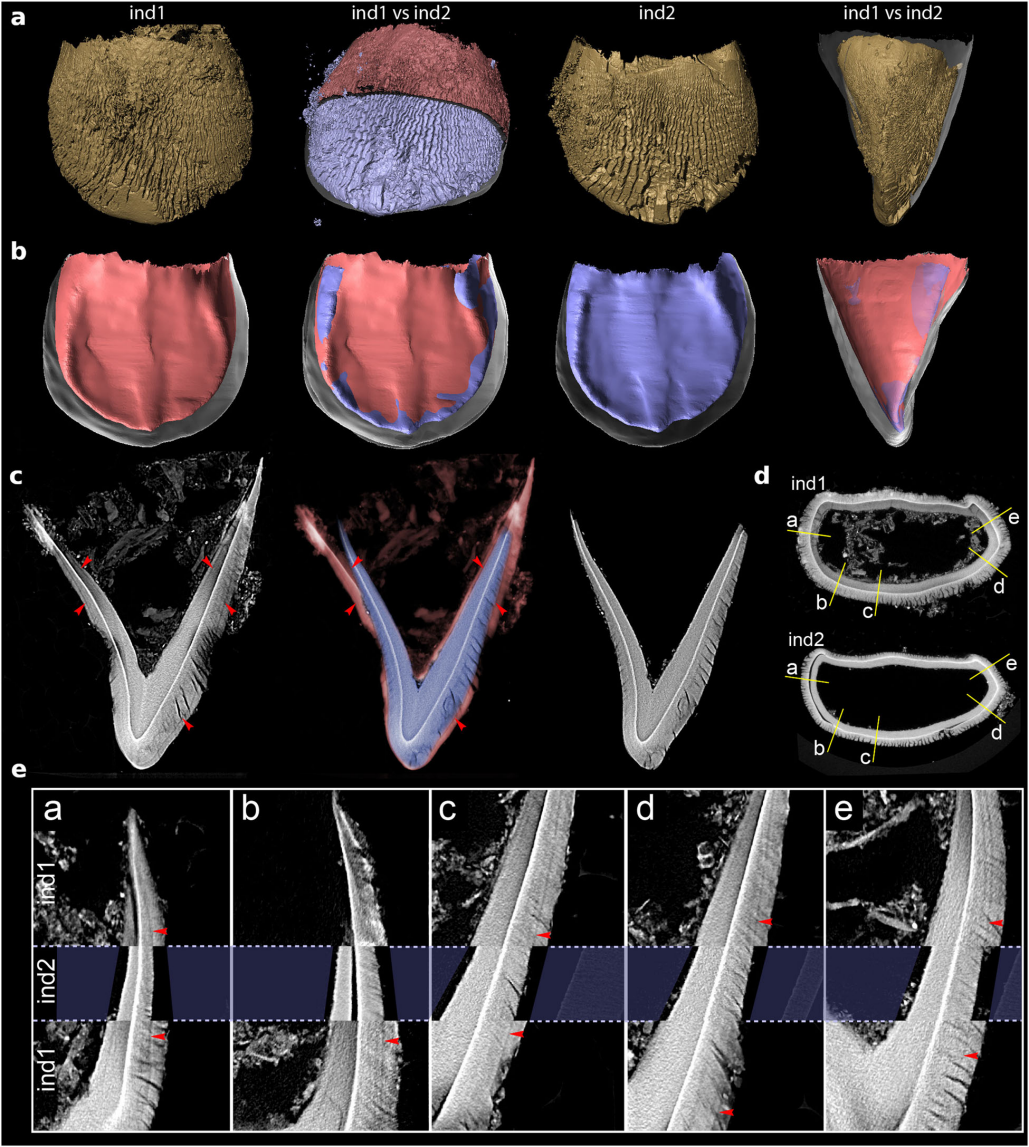
Size comparison of upper second incisors of ind1 and ind2 from µCT data. **a** Labial surface showing enamel degradation. **b** Lingual view of a 3D-reconstruction of the dentinoenamel junction illustrating the similarities between both individuals. From left to right: DEJ of ind1 (red): registration of DEJ surfaces of both individuals; ind2 (blue): comparison of surfaces from a mesiodistal perspective. **c** Neonatal line (NNL) in µCT of ind1 (black rightwards arrowhead), followed by registered overlay of ind2 showing alignment of NNL of ind1 with enamel surface of ind2 (blue) thereby fitting into the area demarcated by the neonatal line of ind1 (red). µCT of ind2 shows no NNL. **d** Horizontal slice showing location of longitudinal slices depicted in panel e. **e** Longitudinal slices at identical positions in ind1 and ind2. Slices are aligned along the dentinoenamel junction. NNL on ind1 (red arrowheads) aligns precisely with enamel surface on ind2 indicating that the tooth size at death of ind2 was the same as that of ind1 at birth.

We further extended the investigation by a morphological inspection of the tympanic plate^27,30,31^. It is evident that the tympanic rings of both individuals are completely isolated, and are not attached to the squamous plate. This process of fixation of the posterolateral segment of the ring to the squamous plate takes place by ca. 35 weeks of gestation^32,33^ and is completed near full term^33^. But due to the fragility and cortical erosions which characterise this region we cannot completely exclude a separation of the rings by taphonomic processes. Thus, based on morphological details and dimensions of the tympanic rings (which differ in their robusticity and dimension) we assume a developmental stage implying a pre- to- near full-term birth, generally defined as period ranging between 36 and 40 prenatal weeks^31,34^. This observation is consistent with data obtained from recent studies which estimate the gestational period in human twins to be between 35 and 37 weeks while the gestational period in singletons is about 40 weeks. However, given the inconsistency of the tympanic plate, and long bone-based age estimations which lack the precision required to identify minor differences in their ages at death, we rely on dental ages as they are not only less variable^35–37^ but, due to their high dentine/enamel densities, less likely to be affected by post-depositional erosive processes. Interestingly, the selected right maxillary second deciduous incisors (i2) of the Krems-Wachtberg infants exhibit generally well-preserved dentine, while enamel was severely degraded in some areas (“Methods” and Fig. 4). Although there is striking similarity in the twins’ i2 morphologies, particularly in the dentinoenamel junction (DEJ) form and size (Fig. 4 and Supplementary Fig. 1), ind1 has larger mesiodistal (MD) and bucco-lingual (BL) dimensions, crown height, enamel and dentin thicknesses and volumes (Supplementary Table 1). These metric differences are consistent with the ones observed for the central incisors, which support the findings based on the lateral incisors. All dimensions indicate a more advanced dental maturation stage for ind1’s teeth (Supplementary Fig. 2, measurement technique see Supplementary Fig. 3). However, several studies of genetic and environmental influences on human dental phenotypic variation based on monozygotic (MZ) twin comparisons report size-specific variations^38,39^, possibly originating in differential prenatal supply. Hence, in the given case, dental size alone cannot be an unambiguous indicator for the age at death estimation.

To obtain further insights into age at death, we considered a more reliable approach^40–42^ and identified the presence/absence of the neonatal line (NNL) within the crown enamel. The NNL, a dark line of Retzius that is only detectable in deciduous teeth and the first permanent molars, reflects dysfunction of the ameloblasts caused by birth stress^43,44^ and separates prenatally and postnatally formed enamel and dentin. Using histological sections and µCT images of the maxillary i2 of all three individuals, an NNL was clearly detectable in ind3 (Supplementary Fig. 4), less distinct in ind1 (here this incremental line runs very close and parallel to the enamel surface), and lacking in ind2 (Fig. 4, Supplementary Fig. 5 and Supplementary Movie 1). We further observed other accentuated lines in the enamel of all individuals, which were most pronounced in ind3.

Though the twins’ teeth showed considerable enamel deterioration, it was possible to obtain more precise age estimates by measuring enamel prism lengths on histological ground sections and in µCT and applying the regression equations for crown formation time^45^. For this purpose, we selected a section in BL direction through the dentine horn in the middle of the teeth (“Methods”). The morphometric results suggest that ind2 died around term (39–40 gestational weeks), ind1 6–7 weeks after birth and ind3 13–14 weeks postpartum (Supplementary Table 2 and Supplementary Fig. 4). It is well known that twin pregnancies are high risk, and are associated with higher prevalence of perinatal mortality of twins than singletons^46,47^. The non-specific stress symptom of subperiosteal newly built bone formations at the tibia of ind1 mentioned above in conjunction with the atypical accentuated lines observed in the enamel emphasise severe stress episodes and/or insufficient supply that might have contributed to the early death of the perinates.

### Chemometrical parameters

#### Stable carbon and nitrogen isotopes

Bulk bone amino acid stable carbon and nitrogen isotopes from ind1 and ind3 are consistent with isotopic dietary evidence for early anatomically modern humans in Europe^48^ and the slightly enriched δ^15^N (0.7‰) and δ^13^C (~0.6‰) values in ind3 are consistent with breastfeeding for a longer duration than ind1 (~10 additional weeks)^49^. Elevated C:N ratios in ind2 indicate post-depositional contamination/degradation of amino acids and the stable isotope measurements for this individual are not reliable (Supplementary Table 3). The available morphometric and stable isotopic results are corroborated by enamel thicknesses and volumes of the i2 crowns measured using µCT images (Supplementary Table 1).

#### Barium distribution in teeth

An alternative approach to detecting a nursing signal and, thus, to uncovering early-life dietary history is to measure barium (Ba)-to-calcium ratios in teeth, and to study the spatial distribution of Ba in enamel which is a biomarker for breastfeeding. Prenatal Ba levels are low because the transfer is restricted by the placenta. In most cases, enrichment occurs after birth by the consumption of mother’s milk. In response to the change of dietary Ba exposure, the incorporated Ba/Ca ratio in enamel should increase at birth and remain elevated for the duration of breastfeeding^50^. This approach is based on the fact that certain elements, such as barium, follow calcium on its transport pathway and are stored in bone and teeth. Barium is absorbed from maternal milk due to its similar chemical properties to calcium^51^. If the Krems-Wachtberg infants survived birth and were breast-fed, a Ba increase in post-NNL enamel would be expected. To assess this nursing signal, we used thin sections of the deciduous second incisors (i2s) and measured ^138^Ba/^43^Ca ratio distributions in dentin, prenatal enamel and postnatal enamel by laser ablation coupled to an inductively coupled plasma quadrupole mass spectrometer (LA ICP-QMS) along with other elements to assess surface contaminations and diagenetic alterations (Supplementary Table 4). Microscopic images after ablation were overlaid with the ^138^Ba/^43^Ca ratios (“Methods” and Supplementary Fig. 6).

**Fig. 5:**
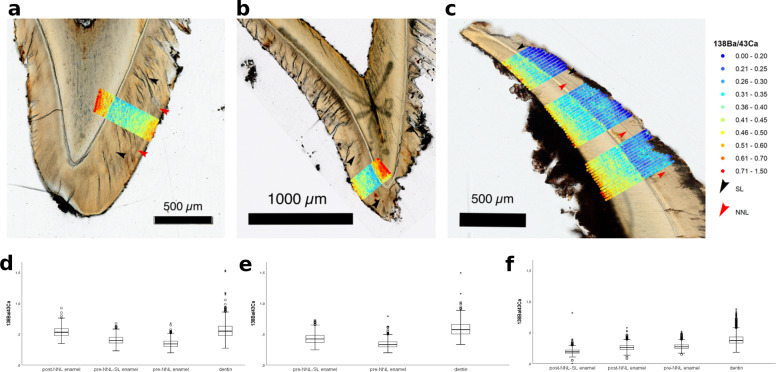
Histologic images displaying sections of ^138^Ba/^43^Ca ratios and statistical evaluation of chemical imaging. **a**–**c** Histologic image and ^138^Ba/^43^Ca ratio sections for individuals ind1, ind2 and ind3, respectively. **d**–**f** Boxplot diagram of statistical evaluation of the ^138^Ba/^43^Ca ratios (cps/cps) in post-NNL enamel, pre-NNL-SL enamel, pre-NNL enamel and dentin (ind1), pre-NNL-SL enamel, pre-NNL enamel and dentin (ind2) and post-NNL-SL enamel, post-NNL enamel, pre-NNL enamel and dentin (ind3) (SL stress line, NNL neonatal line).

The prenatal enamel of ind1 is intersected by an accentuated line. The earliest formed prenatal enamel shows a slightly lower ^138^Ba/^43^Ca ratio than the prenatal enamel formed past the stressline. At the NNL, the ^138^Ba/^43^Ca ratio increases significantly (Fig. 5a, d and Supplementary Table 5) and remains increased to the enamel surface (layer thickness 98 ± 20 µm; the thickness is measured orthogonally to the surface, ±20 µm corresponds to the spot size). Similar distinct features can be observed for ^138^Ba/^31^P and ^88^Sr/^43^Ca ratios (Supplementary Fig. 6g, p). As for ind1, the prenatal enamel of ind2 is intersected by a stress line and the earliest formed prenatal enamel shows a slightly lower ^138^Ba/^43^Ca ratio than the prenatal enamel formed past the stress line (Fig. 5b, e and Supplementary Table 5). It has to be noted that the outermost enamel (49 µm ± 20 µm) of ind2 shows increased ^138^Ba/^43^Ca, ^138^Ba/^31^P, and ^88^Sr/^43^Ca ratios (Fig. 5b, e and Supplementary Fig. 6h, q) similar to post-NNL enamel of ind1, despite the fact that no NNL was recorded for ind2. Ind3 shows no significant increase of ^138^Ba/^43^Ca, ^138^Ba/^31^P and ^88^Sr/^43^Ca ratios in the post-NNL enamel as compared to the pre-NNL enamel. In contrast to this, barium is depleted in the outer layer (45–107 µm) of the post-NNL enamel, indicated by ^138^Ba/^43^Ca, ^138^Ba/^31^P and ^88^Sr/^138^Ba ratios (Fig. 5c, f and Supplementary Fig. 6i, r).

It is generally accepted that human enamel does not undergo significant diagenetic alterations after mineralisation due to its compact structure with little pore space and a minor proportion of organic content (*w* ~ 1%)^50,52–55^. Consequently, it is expected to represent a reliable matrix for biogenic information such as dietary transitions. In addition, the analysed infant teeth were still embedded in the jawbone—and thus additionally protected. Nonetheless, enamel is not immune to diagenetic changes. If the surface is weathered, diffusion might take place resulting in gradients with highest mass fractions in the enamel surface^53,56–58^. Highest ^27^Al/^43^Ca ratios in the enamel layer have been observed in a 10–20-µm-thick layer in the outermost enamel surface of all three individuals, indicating surface contamination. Although age estimation for ind3 clearly implies survival of about 3 months, the ^138^Ba/^43^Ca ratio does not yield a significant difference between pre- and post-NNL enamel (Fig. 5c, f and Supplementary Table 5). However, the change in ^138^Ba/^43^Ca ratios at birth can vary due to inter-individual differences in breastmilk Ba content^50^ or insufficient breastfeeding. The post-NNL enamel of ind3 shows a rudimentary, albeit well developed postnatal incremental line. A decreased ^138^Ba/^43^Ca ratio compared to prenatal and early-postnatal enamel (Fig. 5c, f, Supplementary Fig. 7 and Supplementary Table 5) can be observed from this line. These observations indicate that ind3 passed through further stress episodes during his short postnatal life.

In the case of ind1 and ind2, elevated levels in the outer 10 µm layer are also found for ^138^Ba/^43^C, ^138^Ba/^31^P and ^88^Sr/^43^Ca ratios (Fig. 5a, b and Supplementary Fig. 6g, h, p, q), but no distinct gradients were observed for ^57^Fe/^43^Ca ratios. In the case of significant diagenetic alteration, the penetration depth and corresponding diffusion gradients should be comparable for all three individuals (of a similar age and found within the same sedimentary setting). It is important to note that no significant increase of the ^138^Ba/^43^Ca, ^138^Ba/^31^P and ^88^Sr/^138^Ba ratios can be observed for ind3. Consequently, the increased Ba/Ca levels in ind1 and ind2 at >10–20 µm below the surface cannot be interpreted as diagenetic alteration. Given these considerations, we assume that any diagenetic effects signal from 10 to 20 µm off the surface are of insignificant impact on the biogenic barium (and strontium).

Consequently, the increase in ^138^Ba/^43^Ca, ^138^Ba/^31^P and ^88^Sr/^43^Ca ratios of ind1 starting exactly at the NNL can be interpreted as change of the chemical information and therefore suggest breastfeeding (Fig. 5a and Supplementary Table 5). The ^138^Ba/^43^Ca ratio increases by >30% from prenatal enamel to postnatal enamel, which is consistent with observations on dietary transitions in modern humans^50^. The ^138^Ba/^43^Ca ratio data provide no clear indication/proof for the presence/absence of breastfeeding for ind2. An increased ^138^Ba/^43^Ca level in the outer enamel could indicate a short survival for ind2, although it is nonetheless evident that the layer is significantly thinner when compared to ind1. This indicates an older age at death for ind1.

## Discussion

Confirmed evidence of twins in the archaeological record is extremely rare and has never been verified by aDNA analysis. The present bioanthropological investigation focusses on two infant burials discovered at the Upper Palaeolithic site of Krems-Wachtberg in Lower Austria, one of which represents the remarkable case of a double grave with two infants. By aDNA analysis, we were able to verify them as monozygotic twins while the third infant who had been deposited in the second grave turned out to be their third-degree relative. Based on the construction of the double grave, differential placement of the twins’ corporal remains, and their personalised symbolic treatment, we hypothesised non-synchronous perinatal death and interment and thus a possible re-opening of the grave that had not been backfilled, but covered by a removable construction (mammoth shoulder blade and tusk fragment). By applying morphometric and chemometric approaches for age at death estimation which included analysis of the breastfeeding signal provided by barium, we determined a slightly non-overlapping age at death for the two infants: while ind2 died around pre- to near full-term birth, ind1 most likely survived for about 6–7 weeks. This implies that the inhumation of ind1 required re-opening of the grave. As both individuals are represented as articulated skeletons, the inhumation of ind1 must have occurred at a time when ind2’s soft tissue was not completely degraded. From a forensic point of view, it is likely that the decomposition of the corpse of ind2 had reached the “post bloat” or decay stage^59^. We observed that the body of ind2 was only covered by a millimetre thin layer of red ochre, but not buried in sediment. Although we do not know what impact the red ochre may have had on the soft tissue decay, we can, due to biotic and abiotic environmental factors, assume both delayed decay and desiccation in winter or early spring under the periglacial conditions that prevailed 31,000 years ago. This can explain the observation that no perceivable destruction or damage to ind2’s skeletal remains were caused by the process of re-opening the grave and deposition of ind1. Although personalised symbolic treatment has been documented for burial rituals of the Gravettian^60^, our study shows that Gravettian mortuary behaviour can also include re-opening of a grave and modification/manipulation of its layout and content.

## Methods

### Ancient DNA laboratory work

We took advantage of the recognition that the cochlea of the petrous bones^61^ in most cases yields significantly more endogenous DNA molecules than other skeletal elements^62,63^. The bones used for DNA extraction were digitally preserved through CT scans while the partner petrous bones remain in the original assemblage. The DNA was extracted in dedicated clean room ancient DNA laboratories of the Universities of Vienna and Harvard^64,65^ and was followed by the preparation of libraries using a double-stranded protocol treating some of them with uracil-DNA glycosylase (UDG) to cleave the ancient molecules at uracil bases on the 5′ end, a modification characteristic of ancient DNA, to reduce the rate of errors induced by damage^66^. The DNA libraries were sequenced on a NextSeq500 instrument following in-solution enrichment for sequences overlapping the mitochondrial genome and separately for sequences overlapping ~1.24 million SNPs, and processed bioinformatically as previously described^67^. We assessed evidence for contamination by looking not only at mismatches of the individuals’ mitochondrial DNA to the consensus sequence^68^ but also at heterozygosity on the X chromosome because male individuals only have one copy of the X chromosome^69^. The point estimates varied from 0 to 1.353%, and in conjunction with the high rate of damage in the last nucleotide (ind1: 29.6%; ind2: 10.6%) of the ancient DNA molecules, support the authenticity of the recovered sequences (Table 1).

### *f*-statistics

We merged our newly acquired data for ind1 (Krems1_1) and ind2 (Krems1_2) with previously published data from 5 modern^70–72^ and 73 ancient^24,61,67,73–82^ individuals. Outgroup-*f*_3_ (*qp3Pop*) and *f*_4_-statistics (*qpDstat*) were computed using ADMIXTOOLS. As the outgroup population, we used *Mbuti.DG*, and computed outgroup-*f*_*3*_ statistics of the form *f*_*3*_(*Mbuti.DG*; *Krems1_1/Krems1_2*, *Test*) to investigate the Upper Palaeolithic and Mesolithic individuals with whom Eurasia (*Test*) the Krems-Wachtberg twins shared the highest drift. We also computed statistics of the form *f*_*4*_(*Mbuti.DG*, *Krems1_1/Krems1_2*; *KremsWA3*, *Test*) to evaluate if the Krems-Wachtberg twins shared more alleles with ind3 (*KremsWA3)* than with the same *Test* individuals as before. For the *f*_3_ tests we used default setting and for the *f*_4_-statistics we used the options f4mode: YES and printed: YES. Tests using less than 10,000 SNPs were not considered when analysing the data.

### Morphometric assessment

We selected the right maxillary second deciduous incisors (i2) of ind1 and ind2, and for comparison, a fragment of the right maxilla with embedded tooth germs of ind3’s frontal teeth and measured the i2s based on 3D high-resolution µCT scans and histologic ground sections.

#### Microcomputed tomography (µCT)

High-resolution µCT images of the crowns were obtained with a SCANCO µCT 50 (SCANCO Medical AG, Brüttisellen, CH). All teeth were scanned at a 90 kVp, 0.5 mm Al filter with 1500 projections over 360° with an exposure of 108 µAs. Ind1 and ind2 were reconstructed to 3 µm isotropic resolution. Due to its larger size, the crown of ind3 was scanned with a larger field of view resulting in an isotropic resolution of 4.4 µm.

For all individuals, measurements were performed on the i2, of the crown diameters, height, enamel and dentin thickness and volume. These data were obtained after down sampling all scans to 12 µm isotropic resolution using Fiji 1.51 h^83,84^. The down sampled scans were imported into Amira 6.4 (FEI Thermo Fisher Scientific) and the enamel, dentin and pulp regions were segmented in the segmentation editor using a combination of the lasso tool with autotrace and interpolation between slices. Missing pieces of the enamel and dentin were estimated based on the remaining material (Supplementary Fig. 3a). A surface was generated with the “generate surface” tool (smoothing none, border on, adjust cords selected) and the number of faces was reduced to 5% of the initial number using the simplification editor. Smoothing of the surface was performed applying the “smooth surface” tool (iterations 50, lambda 0.4) (Supplementary Fig. 3b). The DEJ surface area was measured as the interface of enamel and dentin surfaces using the “surface area volume”.

The rim of the tooth germ was estimated as a plane through the cervical line. Sixteen approximately equidistant landmarks were placed along the rim of the crown and a plane (in the following called “cervical plane”) was fitted so that the distance to the landmarks is minimised (Supplementary Fig. 3c). The MD axis direction was aligned along the incisal edge and parallel to the cervical plane. The BL axis direction was placed parallel to the cervical plane and perpendicular to the MD axis direction. The height of the crown was measured as the distance between the cervical plane and a parallel plane which intersects the most coronal point of the crown. The MD and BL diameters were measured between parallel planes at the most extreme points of the crowns along their respective axis directions (Supplementary Fig. 3d).

The coronal 3 mm parallel to the cervical plane of the segmented images were exported as a TIF stack and imported into Fiji 1.51h. The thickness and volume of dentin and enamel were measured with the BoneJ plugin 1.4.2 (ref. ^85^) using the “thickness” and “volume fraction” tools.

For the determination of prism lengths, data sets were analysed in Amira 6.5.0 (FEI Thermo Fisher Scientific) and filtered using built-in Non-local Means filter and Unsharp Masking filter. DEJ turned out to be well preserved in all specimens and was segmented with the segmentation editor. Surfaces of DEJ were aligned to each other and used for registration of image data sets of ind1 and ind2. For prism length measurements, a section in BL direction through the dentine horn in the middle of the tooth (Supplementary Fig. 4a) was virtually cut out (60–180 µm thickness) and visualised as volume rendering with high opacity (Supplementary Fig. 4b, c). Direction of view was oriented along accentuated lines, thereby resulting in an averaging of intensity values in the view direction. Measurements were taken at the labial side of each tooth. Prism length was measured between DEJ starting close to the tip of the dentine horn and the nearest accentuated line. This accentuated line was traced back until it hit the DEJ. From this point, prism length was measured again to the next accentuated line or to the enamel surface, respectively, as described by Birch and Dean^45^.

Prisms were faintly visible in some parts of the virtual sections. Prism orientation was determined by comparison to the corresponding histologic thin ground section, where available. Additionally, ruptures within enamel were used to guide orientation of prism length measurements. In the histologic ground sections, it was possible to observe that these cracks occur predominantly along prisms.

#### Histologic ground sections

Undecalcified histologic thin ground sections oriented in the middle of the tooth in its longitudinal axis were prepared of the second incisors of ind1, ind2 and ind3 (ref. ^86^). For ind3 the section was carried out through the maxillary fragment hereby cutting the second incisor. Specimens were ground stepwise to a thickness of first 400 µm, then 250 µm and finally 100 µm and scanned at every step with an Olympus BX61VS digital virtual microscopy system (dotSlide 2.4; Olympus Tokyo, Japan), with a resolution of 0.32 µm/pixel. The final 100-µm-thick slides remain as a permanent record and were also used to determine the spatial distribution of Barium.

Poor enamel preservation did not allow direct daily increment count in histologic ground sections and were therefore measured in a similar way as µCT images, using the method described by Birch and Dean^45^. Histologic images were analysed and measured independently by a different observer than µCT images.

### Chemometric analyses

#### Stable carbon and nitrogen isotopes

Collagen in the Krems-Wachtberg bone samples was poorly preserved and pre-treated using a modified XAD process to extract and purify amino acids^87^. Samples were demineralised in 0.5 N HCl for 2–3 days at 5 °C. The demineralised collagen pseudomorph was gelatinised at 60 °C in 1–2 mL 0.01 N HCl for 8–10 h. Sample gelatin was pipetted into a pre-cleaned 10 mL disposable syringe with an attached 0.45 mm Millex Durapore PVDF filter (pre-cleaned with methanol and Nanopure H_2_O) and driven into a thick-walled culture tube. The filtered solution was then lyophilised, and percent gelatinisation and yield determined by weight. The sample gelatin was then hydrolysed in 2 mL 6 N HCl for 22 h at 110 °C. Supelco ENVI-Chrom® SPE (Solid Phase Extraction; SigmaeAldrich) columns were prepped with two washes of methanol (2 mL) and rinsed with 10 mL DI H_2_O. With a 0.45 mm Millex Durapore filter attached, the SPE Column was equilibrated with 50 mL 6 N HCl and the washings discarded. Two milliliters of collagen hydrolysate as HCl was pipetted onto the SPE column and driven with an additional 10 mL 6 N HCl dropwise with the syringe into a 20-mm culture tube. The hydrolysate was finally dried into a viscous syrup by passing UHP N2 gas over the sample heated at 50 °C for ~12 h. Carbon and nitrogen concentrations and stable isotope ratios of the XAD amino acid samples were measured at the Yale Analytical and Stable Isotope Center with a Costech elemental analyser (ECS 4010) and a Thermo DeltaPlus analyzer. Sample quality was evaluated by % crude gelatin yield, %C, %N and C:N ratios^88^. C:N ratios for ind1 and ind3 fall between 2.9 and 3.6, indicating good collagen preservation^89^. The C:N ratio for ind2 falls outside this range and the measurements are unreliable.

### Ba chemometric analysis

The LA ICP-QMS measurements to detect the spatial distribution of ^138^Ba/^43^Ca ratios of the thin sections of the i2s from ind1, ind2 and ind3 were performed using a nanosecond excimer-based laser ablation system (NWR 193, ESI-NWR Division, Electro Scientific Industries, Inc., Portland, CA, USA) coupled to an ICP-QMS (NexION 350D, Perkin Elmer, Waltham, MA, USA) similar to the protocol of Draxler et al.^90^. Further multi-elemental distributions were monitored to define the tooth tissue (^31^P) and to identify diagenetic alterations (^27^Al, ^57^Fe, ^88^Sr). The laser ablation was performed in line scans from the dentin towards the enamel. Prior of each line, a gas blank of 10 s was measured. In case of ind1 and ind2, one area of well-preserved enamel towards the crown was ablated using 10 adjacent lines. Additional single lines were ablated for evaluation of diagenetic alterations. For ind3, the area close to the labial apical crown edge with a visible NNL was ablated. An in-house pressed pellet of the reference material NIST SRM 1486 (bone meal, NIST, Gaithersburg, USA)—certified for P, Ca and Sr; in-house determined value of Ba (281 µg g^−1^ ± 40 µg g^−1^ (SD, *n* = 6))—was measured using line measurements under same conditions as the samples at the beginning and end of each laser ablation event for quantification. General instrumental settings for the LA ICP-QMS measurement are summarised in Supplementary Table 4.

The mean gas blank values (62 data points) were subtracted from the measured values of the samples for each line. The limit of detection (LOD) was calculated as 3× standard deviation (SD) of this blank signal. Data reduction was performed with an in-house developed MS Excel Macro^90^. All blank corrected values smaller than the LOD were set to zero. The ^138^Ba values were normalised to ^43^Ca signals. Based on the Ca-normalised intensities, the mass fractions of Ba were determined applying a one-point-calibration using certified reference material NIST SRM 1486.

Microscope images of the thin sections of the i2s from ind1, ind2 and ind3 were taken after the laser ablation including a spatial scale into the image using a standard binocular microscope (S63T Trinocular Pod 8–50×) connected to a digital camera (ProgRes CT3, Jenoptik, Jena, Germany) in 8- to 10-fold magnification. The microscope and histological images were imported and georeferenced in the geographical mapping software ArcGIS® 10.4.1 (ESRI, Redlands, CA, USA) using the spatial reference scale to create a spatial reference system for the images. The laser ablation lines were digitised, and the *x*–*y* coordinates of start and end point of each line were determined. With the knowledge of the distance between the first and last data point in *x* and *y* direction, and the amount of data points in between, the in-house developed MS Excel Macro calculated the intermediate *x*- and *y*-coordinates for each data point (*z*-value), according to Draxler et al.^90^. These data (including multi-elemental data and ratios) were than imported to ArcGIS® as an overlay to histological images. Chemical images for the distribution of ^138^Ba/^43^Ca, ^138^Ba/^31^P, ^27^Al/^43^Ca, ^88^Sr/^138^Ba and ^88^Sr/^43^Ca ratios were created. Further details on the creation of spatial images using the software tools of ArcGIS® are published elsewhere^91–93^.

#### Selection of regions of interest (ROI)

ROIs of pre-NNL enamel, post-NNL enamel and dentin were histologically identified on the microscope images and digitalised using ArcGIS® for further (statistical) interpretation of the data sets. In addition, stress lines were identified and digitalised using ArcGIS®. The stress lines were used to subdivide the pre-NNL and post-NNL enamel. The digitalised ROIs were intersected with multi-elemental data and ratios and exported for further statistical evaluation. The export of the measured and normalised data of the corresponding ROIs from ArcGIS® allowed for a comparative spatial statistical evaluation of the data, and the associated ROIs by using PASW 18®. Boxplots for the selected ROIs and overview tables of percentiles (5%, 25%, 75%, 95%, median, mean and standard deviation) were created (Fig. 5d–f and Supplementary Table 5).

#### Chemometric thickness estimation of post-NNL enamel – ind1 and ind2

The boundary of the layer with increased ^138^Ba/^43^Ca ratios (~0.41–1.5) in enamel was determined by converting the number of enamel data points above the maximum prenatal ^138^Ba/^43^Ca ratio into a distance (using instrumental integration time and laser ablation speed). The maximum prenatal ^138^Ba/^43^Ca ratio was defined by the mean ^138^Ba/^43^Ca ratio plus three times the standard deviation in the region adjacent to DEJ, corresponding to the material developed before birth.

### Statistics and reproducibility

The statistical methodologies used are described above in this section. Genetic analysis, namely kinship assessment, was performed independently by two of the researchers.

### Reporting summary

Further information on research design is available in the Nature Research Reporting Summary linked to this article.

## Supplementary information

Supplementary Information

Description of Additional Supplementary Files

Supplementary Data 1 and 2

Supplementary Movie 1

Reporting Summary

## Data Availability

The raw data are available at the European Nucleotide Archive with the accession number PRJEB40336.

## Code availability

All custom code related to the ancient DNA analysis is available at https://github.com/DReichLab/ADNA-Tools. The Excel Macro used for the chemometric analysis is available at https://oc.unileoben.ac.at/index.php/s/pCA8WIMntQZfIDf.
