# Supplementary material for: Ancient DNA reveals monozygotic newborn twins from the Upper Palaeolithic
Source: Commun Biol. 2020 Nov 6;3:650. doi: 10.1038/s42003-020-01372-8 (PMC7648643; doi:10.1038/s42003-020-01372-8)
Supplement: Supplementary file 1 — Supplementary Information [file 42003_2020_1372_MOESM1_ESM.pdf]

Supplementary Information

Ancient DNA reveals monozygotic newborn twins from the Upper Paleolithic

Teschler-Nicola et al.

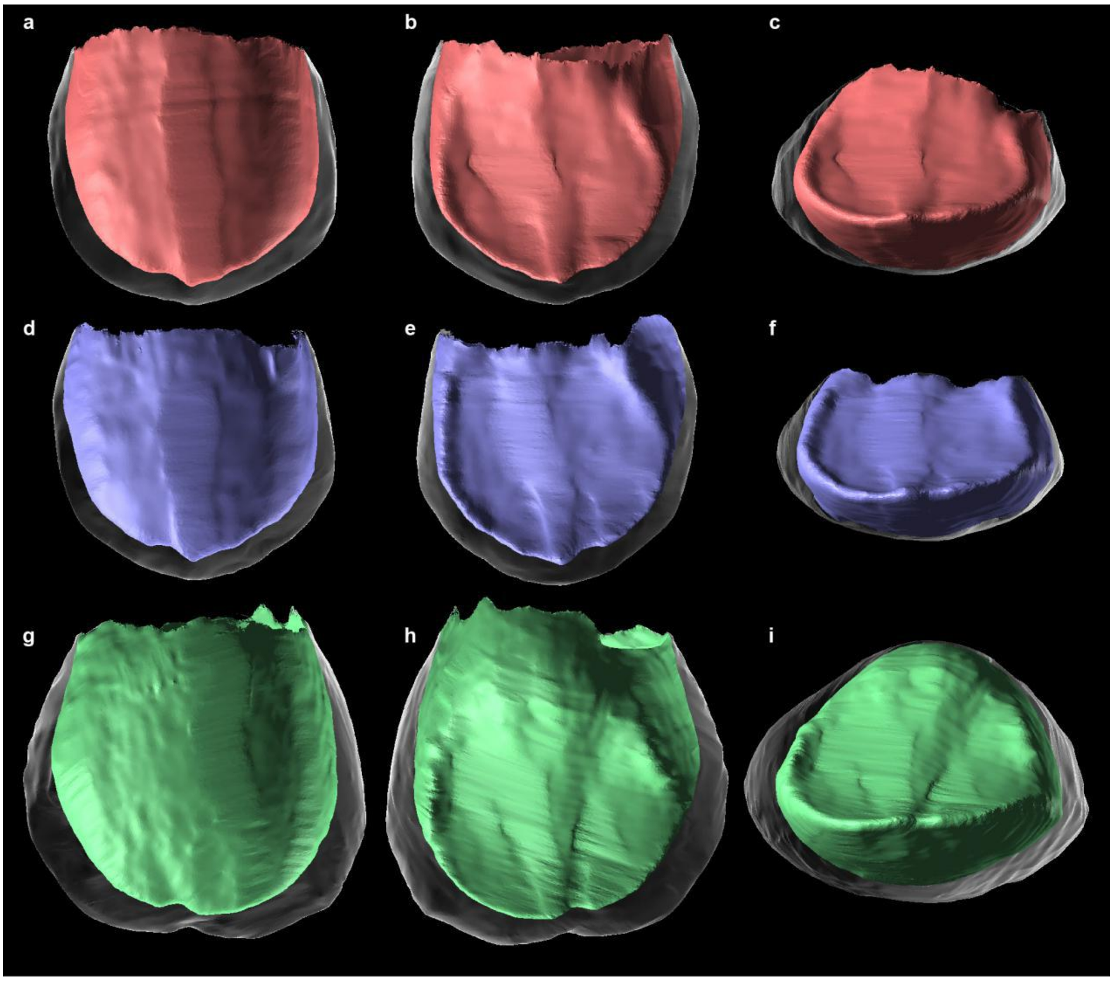

Supplementary Fig. 1: Morphology of maxillary right second deciduous incisors (i2). Ind1 in the first row (**a-c**), ind2 in the second row **(d-f)** and ind3 in the third row **(g-i)**. Shown are the dentinoenamel junction in colour with transparent enamel surface. **a,d,g**, Labial perspective. **b,e,h,** Lingual perspective. **c,f,i**, Occlusal perspective.

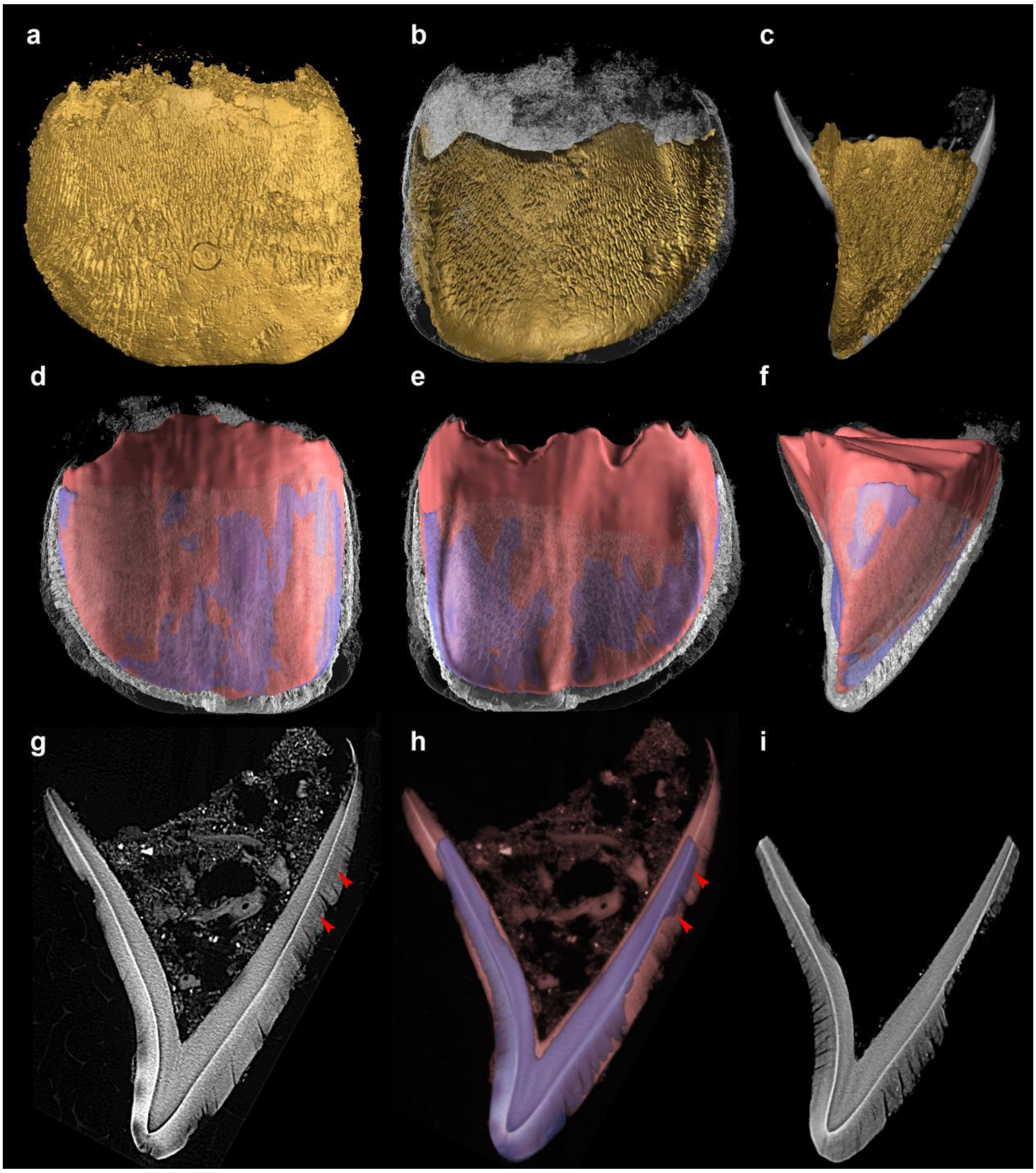

Supplementary Fig. 2: **Size comparison of the maxillary right first deciduous incisor (i1) of ind1 and ind2 from registered µCT data. a,** Labial surface of the enamel of ind1 showing degree of degradation. **b,** Lingual surface of the enamel of ind2; surface of ind1 is shown in white. **c,** Distal enamel surface of ind2 with slice through ind1. **d,e,f,** Dentinoenamel junction of ind1 (red) and ind2 (blue) from labial, lingual and distal direction. Enamel surface is shown in white. **g,** Slice through ind1 showing faint neonatal line (NNL) (➤). **h,** Overlay of ind1 and ind2. NNL (➤) on ind1 aligns with enamel surface on ind2. **i,** slice through ind2 at same position as panel g.

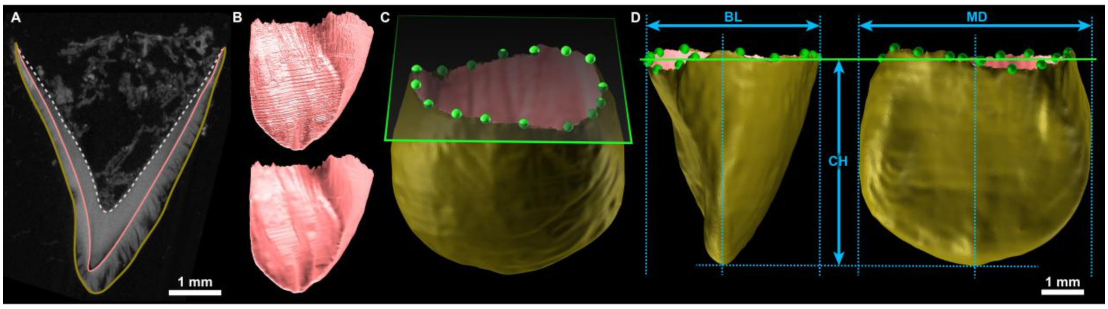

Supplementary Fig. 3: Tooth measurements on µCT scans. A, Enamel surface, dentinoenamel junction (DEJ) and dentine surface. **B,** DEJ without and with smoothing. **C,** Landmarks at the crown rim with fitted cervical plane. **D,** Crown height (CH) and diameters in buccolingual (BL) and mesiodistal (MD) direction.

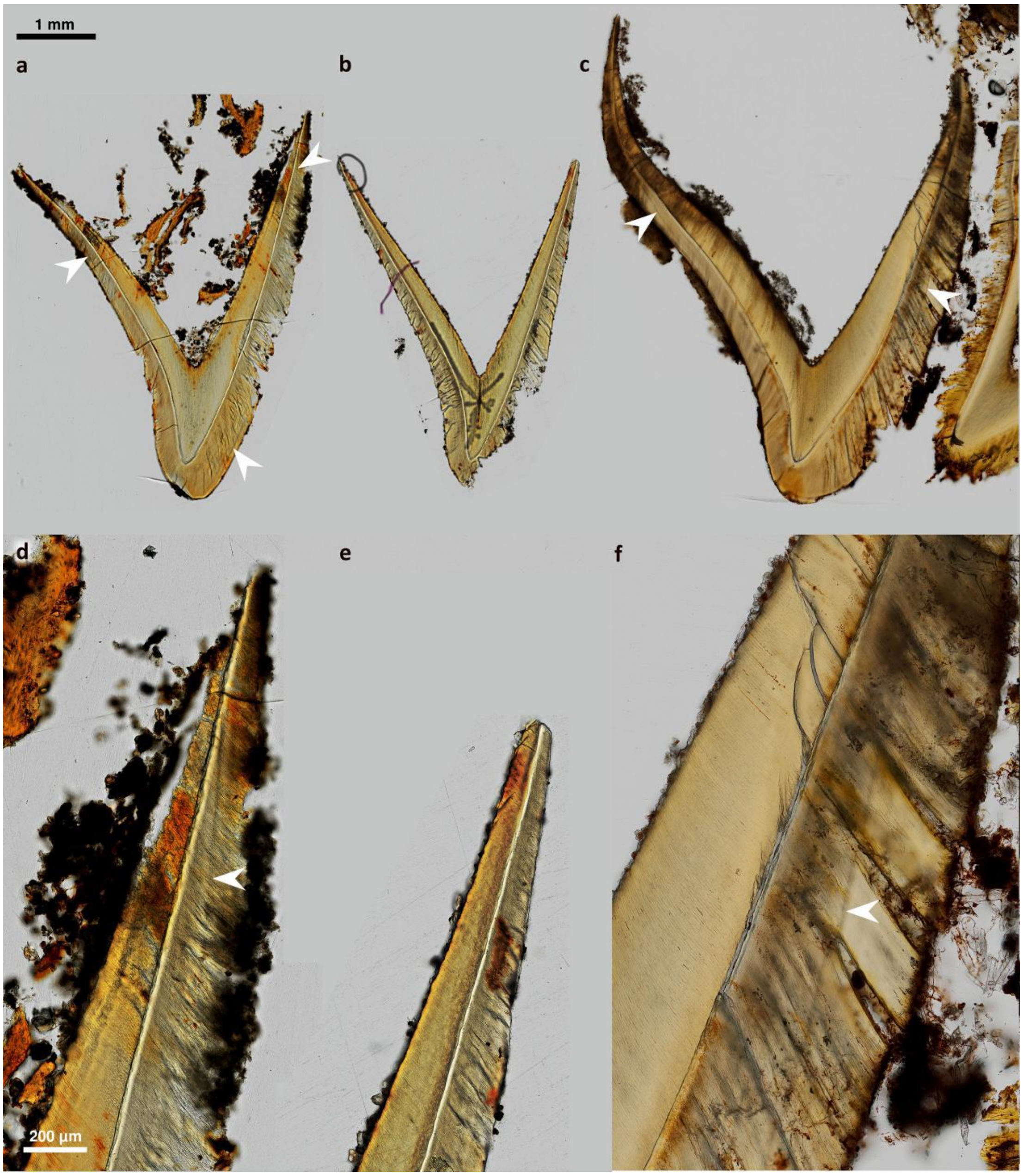

Supplementary Fig. 4: Micrographs of the upper lateral incisors (i2) Histology of ind1. **(a,d)**, Histology of ind2. **(b,e)**, Histology of ind3. **(c,f)**. Magnified histological details are depicted below **(d,e,f)**. Neonatal lines (arrowheads) are visible in ind1. **(d)** and ind3. **(f)** but missing in ind2. **(e).**

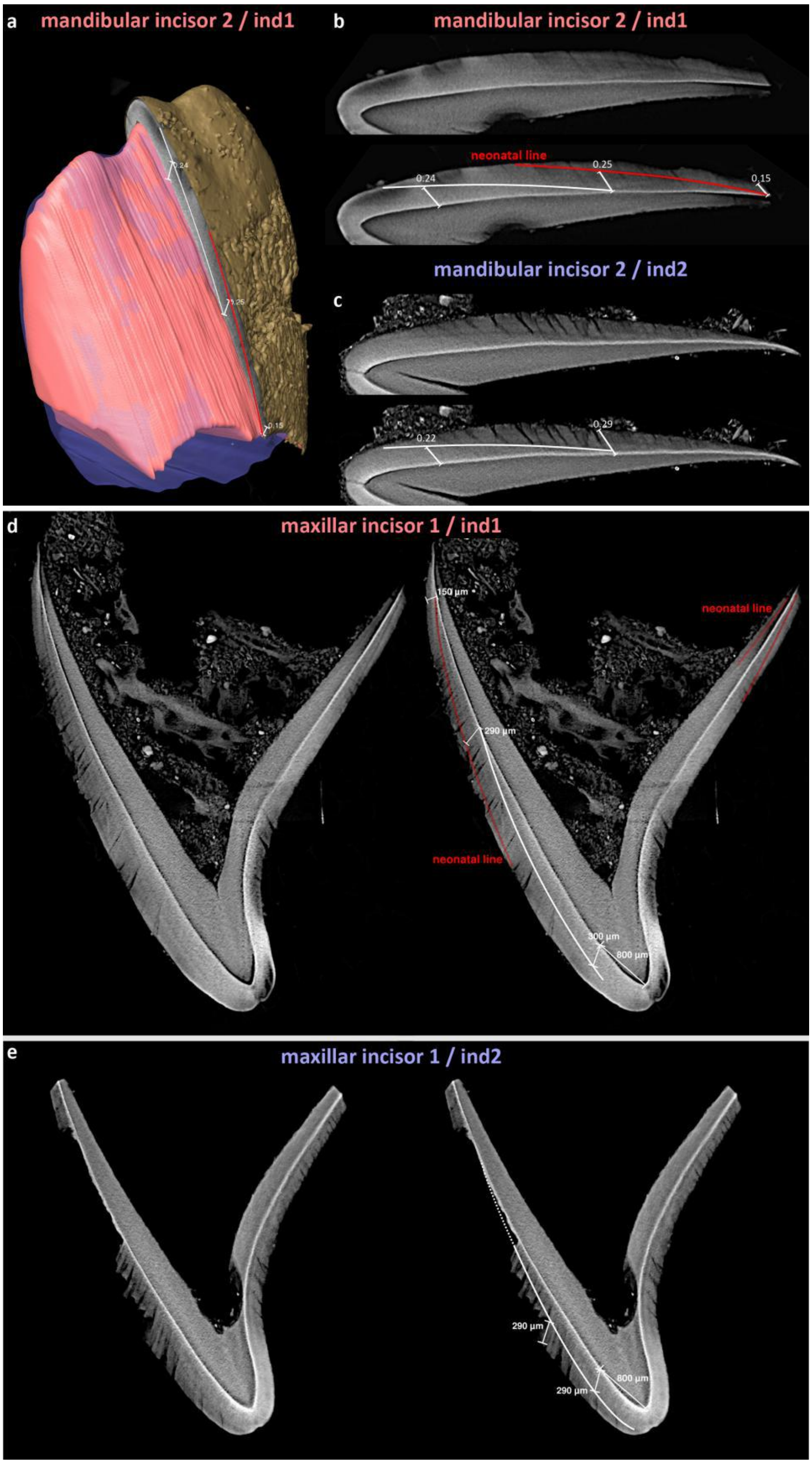

Supplementary Fig. 5: Prism length measurements on µCT scans. a, Mandibular i2 of ind1, overview showing position of measurements on a cutting plane in buccolingual direction through the dentine horn in the middle of the tooth. **b,** Mandibular i2 of ind1, virtual section displayed as volume rendering; without and with measurements. **c,** Mandibular i2 of ind2, virtual section displayed as volume rendering, without and with measurements. **d,** Maxillar i1 of ind1, virtual section displayed as volume rendering; without and with measurements. **e,** Maxillar i1 of ind2, virtual section displayed as volume rendering, without and with measurements. Neonatal line in red and accentuated lines in white.

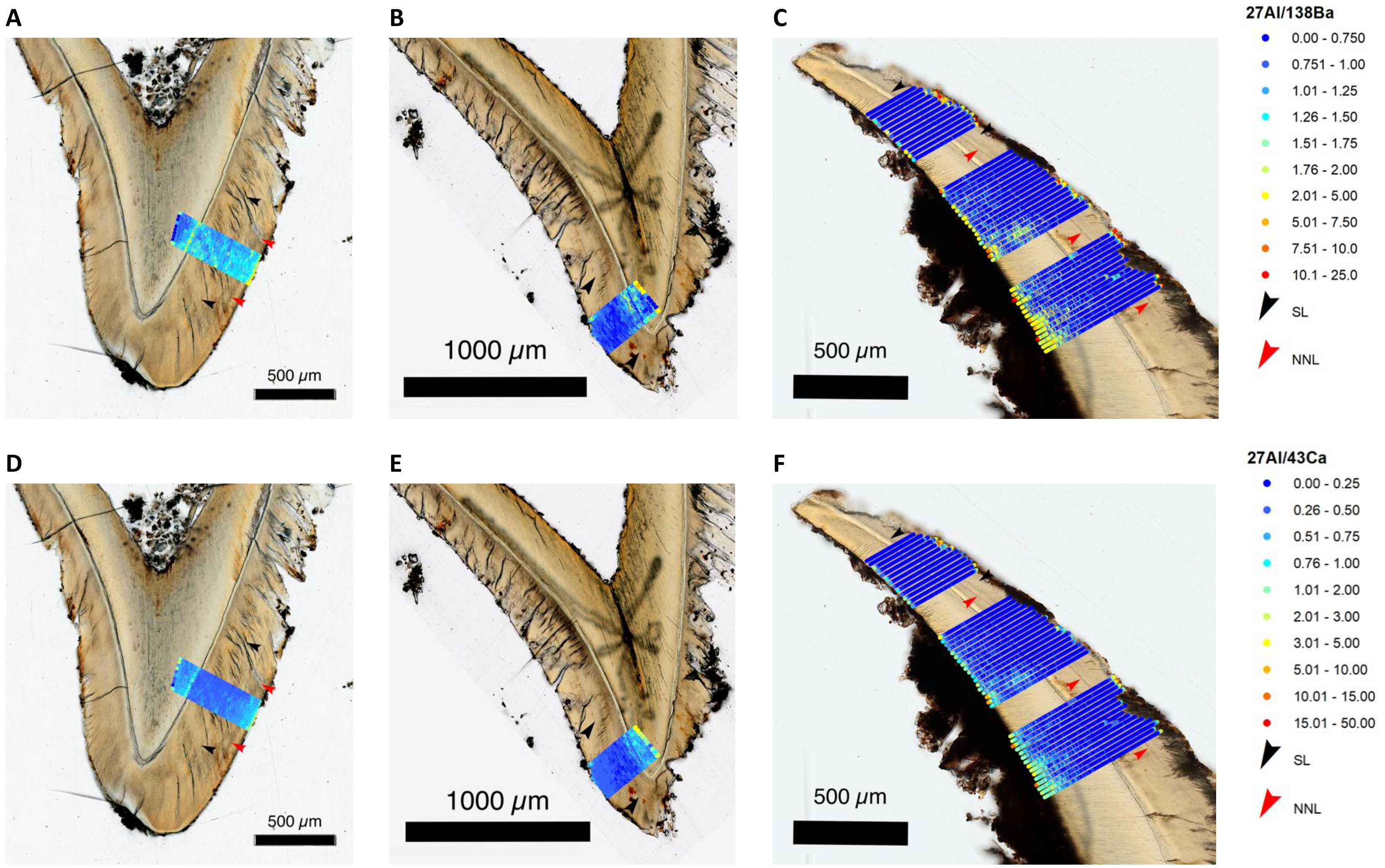
A
500 μm
B
1000 μm
C
500 μm
27Al/138Ba
0.00 - 0.750
0.751 - 1.00
1.01 - 1.25
1.26 - 1.50
1.51 - 1.75
1.76 - 2.00
2.01 - 5.00
5.01 - 7.50
7.51 - 10.0
10.1 - 25.0
SL
NNL
D
500 μm
E
1000 μm
F
500 μm
27Al/43Ca
0.00 - 0.25
0.26 - 0.50
0.51 - 0.75
0.76 - 1.00
1.01 - 2.00
2.01 - 3.00
3.01 - 5.00
5.01 - 10.00
10.01 - 15.00
15.01 - 50.00
SL
NNL

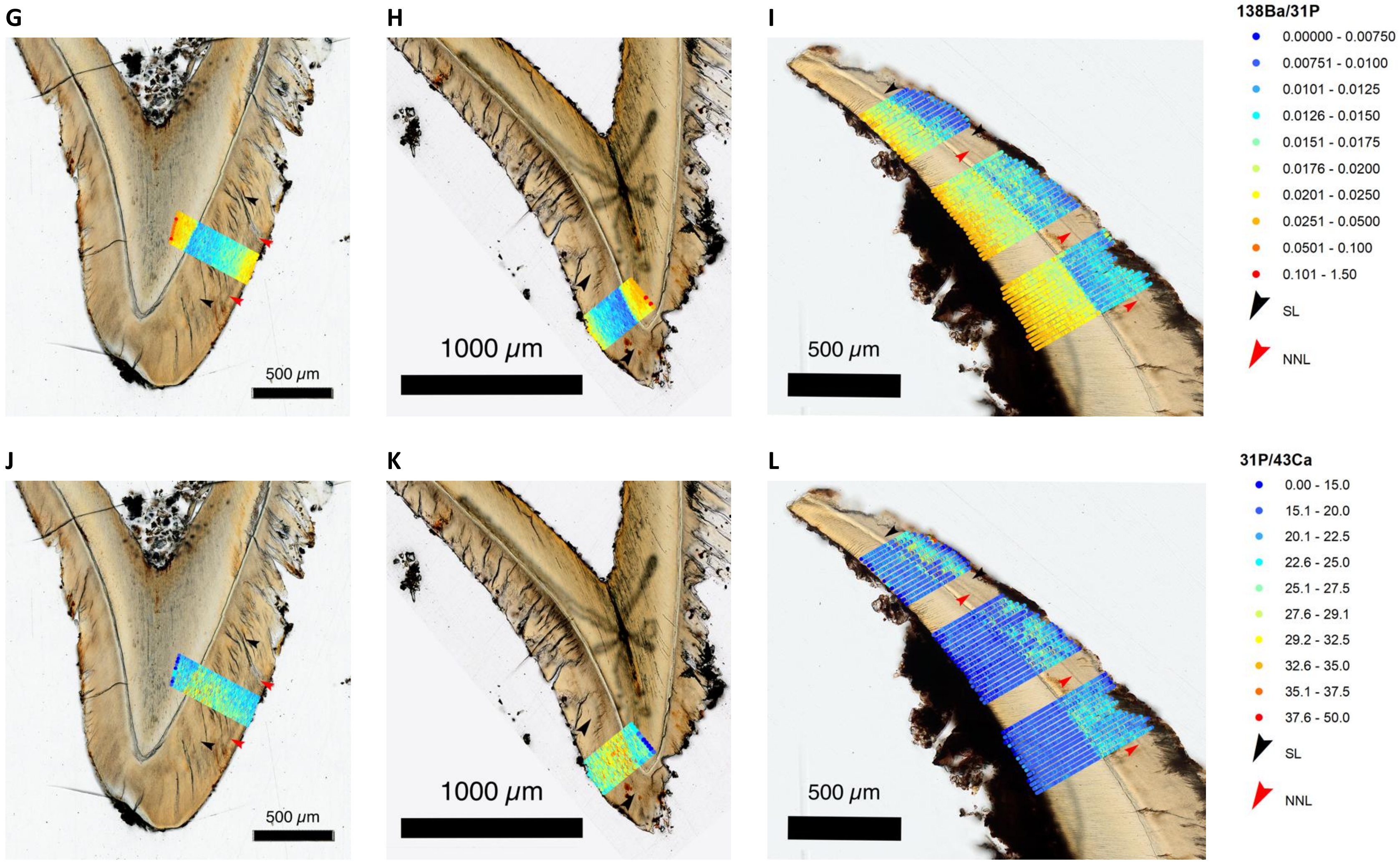
G
H
I
500 μm
1000 μm
500 μm
138Ba/31P
0.00000 - 0.00750
0.00751 - 0.0100
0.0101 - 0.0125
0.0126 - 0.0150
0.0151 - 0.0175
0.0176 - 0.0200
0.0201 - 0.0250
0.0251 - 0.0500
0.0501 - 0.100
0.101 - 1.50
SL
NNL
J
K
L
500 μm
1000 μm
500 μm
31P/43Ca
0.00 - 15.0
15.1 - 20.0
20.1 - 22.5
22.6 - 25.0
25.1 - 27.5
27.6 - 29.1
29.2 - 32.5
32.6 - 35.0
35.1 - 37.5
37.6 - 50.0
SL
NNL

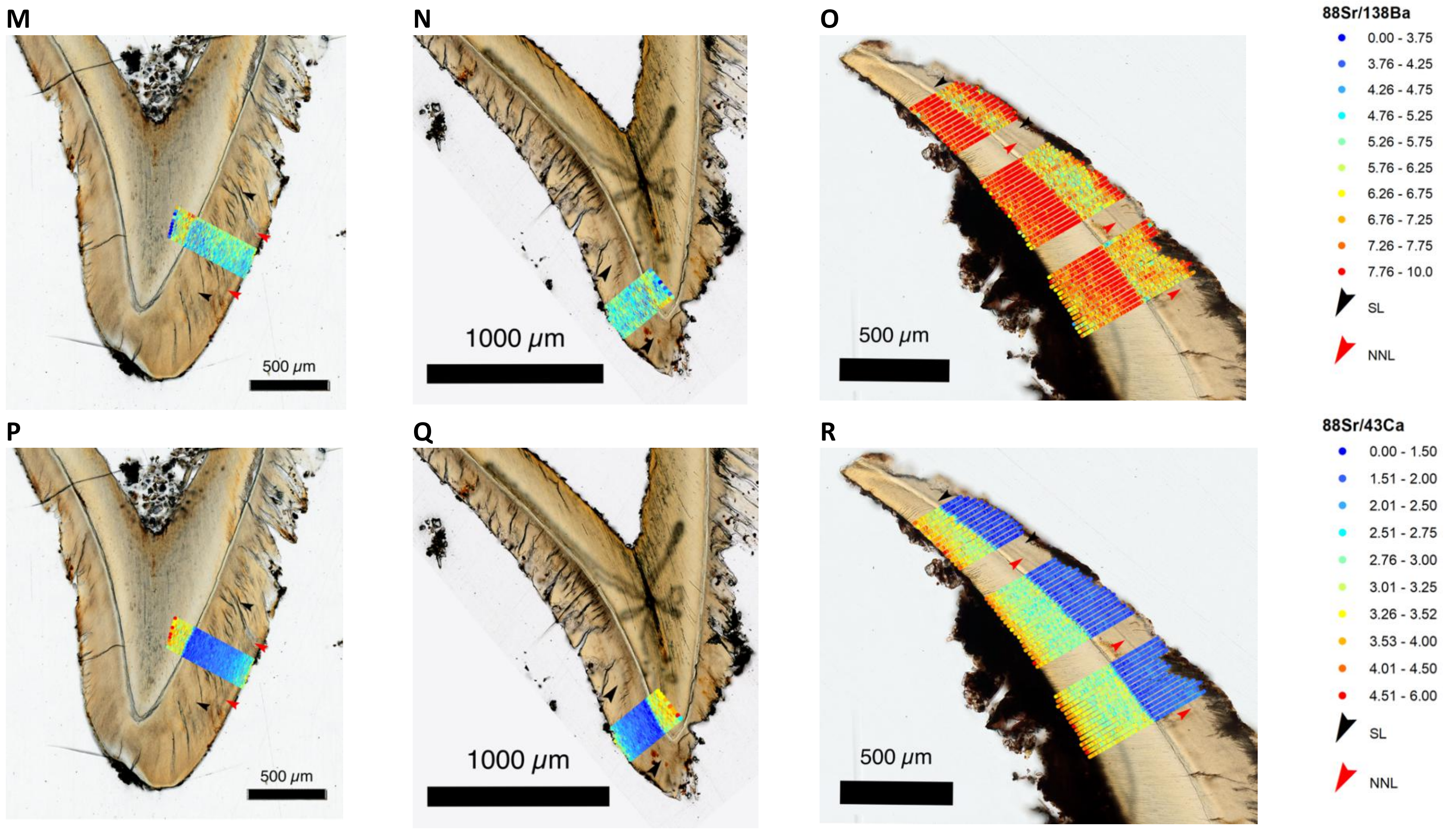

Supplementary Fig. 6: Histologic images and sections of elemental ratios, and statistical evaluation of chemical imaging. A, B, C, Histologic image and $^{27}Al/^{138}Ba$ ratio sections for individuals ind1, ind2, and ind3, respectively. **D, E, F,** Histologic image and $^{27}Al/^{43}Ca$ ratio sections for individuals ind1, ind2, and ind3, respectively. **G, H, I,** Histologic image and $^{138}Ba/^{31}P$ ratio sections for individuals ind1, ind2, and ind3, respectively. **J, K, L,** Histologic image and $^{31}P/^{43}Ca$ ratio sections for individuals ind1, ind2, and ind3, respectively. **M, N, O,** Histologic image and $^{88}Sr/^{138}Ba$ ratio sections for individuals ind1, ind2, and ind3, respectively. **P, Q, R,** Histologic image and $^{88}Sr/^{43}Ca$ ratio sections for individuals ind1, ind2, and ind3, respectively.

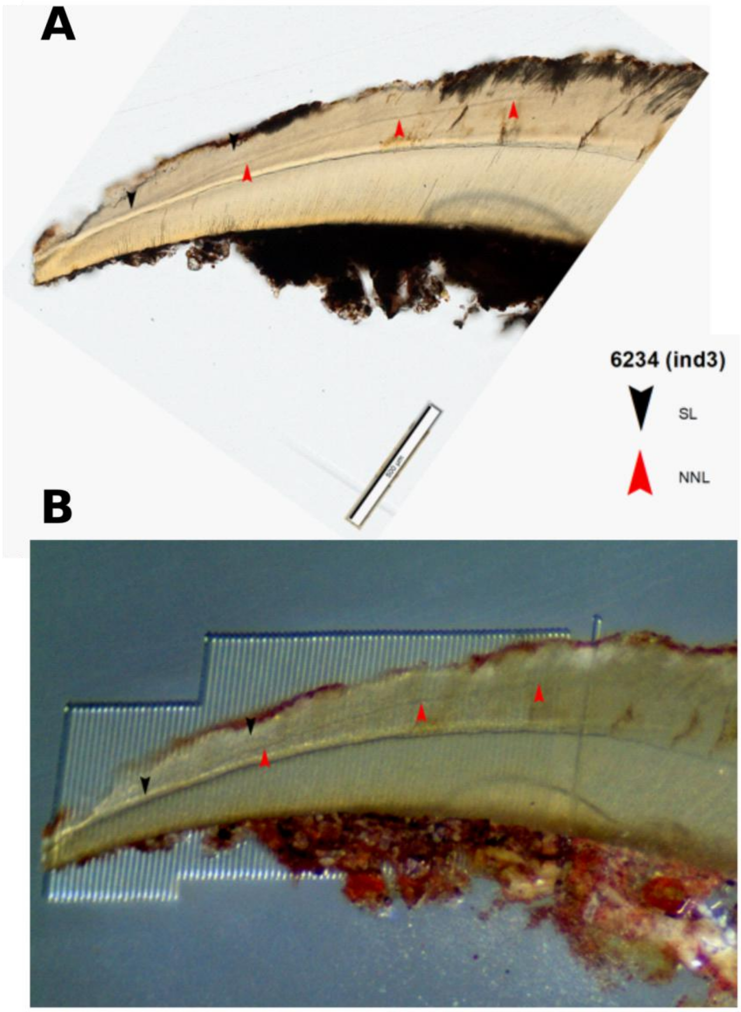

Supplementary Fig. 7: Histologic and microscopic images of ind3. A Histologic image of ind3 with indication arrows for neonatal-line (NNL) and post-NNL stress line (SL). **B** Microscopic image past laser ablation of ind3 with indication arrows for neonatal line (NNL) and post-NNL stress line (SL).

Supplementary Table 1: Metric dimensions of the right maxillary second deciduous incisor of the Upper Palaeolithic Krems-Wachtberg infants: individual 1 (ind1), individual 2 (ind2) and individual 3 (ind3). Thickness and volume measurements are limited to the most apical 3 mm of the teeth.

		ind1	ind2	ind3
**Extent**	**Mesial-Distal**	5.418	5.316	6.367
(mm)	**Buccal-Lingual**	4.038	3.292	4.813
	**Crown Height**	4.858	4.594	6.013
**Thickness**	**Dentin**	0.375	0.324	0.514
(mm)	**Enamel**	0.385	0.322	0.570
**Volume**	**Dentin**	5.401	4.461	7.519
(mm^3)	**Enamel**	9.663	7.289	16.012

Supplementary Table 2: Results of age estimation applying the regression equations of Birch and Dean (2014). Upper and lower incisors were analysed using histologic specimens or µCT scans. Measurements of enamel prism length between Crown initiation (Ci), Accentuated Line (AL1), Neonatal line (NNL) and Crown end (Ce) are presented in micrometre. Results include age at death for all individuals and postnatal survival time for ind1 and ind3.

			Prism Length (µm)			Age	Postnat. Survival	
			Ci AL1 NNL Ce			(weeks)	(weeks)	(days)
ind1	i1 maxilla	µCT	300	290	150	45.2	6.3	44
	i2 maxilla	µCT	280	250	170	46.3	7.2	50
	i2 maxilla	histology	525		178	46.4	7.5	53
	i2 mandible	µCT	240	250	150	44.0	6.4	45
ind2	i1 maxilla	µCT	290	290		39.5	-	-
	i2 maxilla	µCT	280	240		39.4	-	-
	i2 maxilla	histology	216	262		37.9	-	-
	i2 mandible	µCT	220	290		39.1	-	-
ind3	i1 maxilla	µCT	650		320	53.3	12.9	90
	i2 maxilla	µCT	660		320	56.9	12.9	90
	i2 maxilla	histology	673		339	56.1	13.6	95
	i2 mandible	µCT	671		327	55.5	13.1	92

Supplementary Table 3: Results of the N- and C-isotope ratios.

Provenience	Sample ID	weight	d $^{15}N_{AIR}$	d $^{13}C_{VPDB}$	%N	%C	C:N
Austria; Krems-Wachtberg; ind3	150025	0.980	12.6	-19.9	2.7	7.8	3.30
Austria; Krems-Wachtberg; ind1; inv. No. 151425+151435	151566	0.900	11.9	-20.5	2.3	6.6	3.36
Austria; Krems-Wachtberg; ind2; inv. No. 151368+151461	151567	1.140	-1.6	-24.1	0.2	0.9	5.60**

* error on all reported measurements is 0.2

**elevated C:N ratio indicates the isotopic measurements are unreliable.

Supplementary Table 4: Operating conditions of the laser ablation ICP-QMS setup.

**Operating conditions**	
LA parameters (New Wave NWR 193)	
laser wavelength	193 nm
spot diameter	20 µm
line spacing	0 µm
scan speed	10 µm/s
wash time between lines	20 s
frequency	10 Hz
laser energy	60 %
carrier gas	He
carrier gas flow rate	0.9 L min^{-1}
ICP-QMS parameters (Perkin Elmer NexION 350D)	
ICP RF power	1300 W
gas	Ar
auxiliary gas flow rate	0.75 L min^{-1}
plasma gas flow rate	15 L min^{-1}
sampler cone	nickel
skimmer cone	nickel
detector mode	dual
measured isotopes	^{27}Al, ^{31}P, ^{138}Ba, ^{43}Ca, ^{88}Sr<br>ind1 and ind2: additionally ^{57}Fe
integration time	0.176 s
dwell time	30 ms

Supplementary Table 5: Statistical evaluation of chemical imaging. $^{138}Ba/^{43}Ca$ ratios (cps/cps) and Ba mass fraction (µg g^{-1}) given as percentiles (5%, 25%, 75%, 95%, median, mean and standard deviation) in post-SL enamel, post-NNL enamel, pre-NNL enamel and dentin for ind1, ind2 and ind3.

		$^{138}Ba/^{43}Ca$ (cps/cps)							Ba (µg g^{-1})						
		Percentile 05	Percentile 25	Median	Percentile 75	Percentile 95	Mean	SD	Percentile 05	Percentile 25	Median	Percentile 75	Percentile 95	Mean	SD
ind1	post-NNL enamel	0.42	0.47	0.53	0.59	0.68	0.54	0.08	207	234	262	291	335	264	42
	pre-NNL-SL enamel	0.31	0.36	0.40	0.45	0.52	0.40	0.07	151	175	197	221	256	199	33
	pre-NNL enamel	0.25	0.30	0.34	0.39	0.46	0.35	0.07	123	148	168	190	226	171	32
	dentin	0.38	0.48	0.55	0.63	0.80	0.59	0.42	188	235	270	310	396	291	207
ind2	pre-NNL-SL enamel	0.31	0.37	0.42	0.48	0.60	0.43	0.09	152	181	208	236	295	212	43
	Pre-NNL enamel	0.24	0.30	0.33	0.38	0.45	0.34	0.07	119	146	163	185	224	167	33
	dentin	0.43	0.50	0.57	0.66	0.82	0.79	3.13	210	248	282	324	402	387	1540
ind3	post-NNL-SL enamel	0.14	0.17	0.19	0.22	0.27	0.20	0.05	70	82	93	107	131	96	23
	post-NNL enamel	0.18	0.22	0.26	0.29	0.34	0.26	0.05	87	110	127	142	169	127	25
	pre-NNL enamel	0.21	0.24	0.27	0.30	0.35	0.27	0.04	103	120	133	149	173	135	22
	dentin	0.29	0.33	0.37	0.43	0.54	0.39	0.08	141	162	183	211	266	190	39
