# Supplementary material for: Ancient DNA reveals monozygotic newborn twins from the Upper Palaeolithic
Source: Commun Biol. 2020 Nov 6;3:650. doi: 10.1038/s42003-020-01372-8 (PMC7648643; doi:10.1038/s42003-020-01372-8)
Supplement: Supplementary file 2 — Description of Additional Supplementary Files [file 42003_2020_1372_MOESM2_ESM.pdf]

Description of Additional Supplementary Files

File Name: Supplementary Movie 1 Description: Tracking shot using micro-CT scans through the central parts of the upper lateral incisors (i2) ind1 to the left, ind2 to the right. Red arrows mark the neonatal lines and white arrows mark accentuated lines.

File Name: Supplementary Data 1 Description: Sequencing data details for the two newly reported samples (ind1 and ind 2) and the previously reported KremsWA3 individual (ind3). (as Excel file)

File Name: Supplementary Data 2 Description: Results of the tests f4(Mbuti.DG, Krems1_1/Krems1_2; KremsWA3, Test) and f3(Mbuti.DG; Krems1_1/Krems1_2/KremsWA3, Test). These results were used to produce panels A) and B) of Fig. 2, respectively.
